# Conserved expression of vertebrate microvillar gene homologs in choanocytes of freshwater sponges

**DOI:** 10.1186/s13227-016-0050-x

**Published:** 2016-07-12

**Authors:** Jesús F. Peña, Alexandre Alié, Daniel J. Richter, Lingyu Wang, Noriko Funayama, Scott A. Nichols

**Affiliations:** Department of Biological Sciences, University of Denver, F.W. Olin Hall, Room 102, 2190 E. Iliff Ave., Denver, CO 80208 USA; Laboratoire de Biologie du Développement de Villefranche-sur-mer, CNRS, Sorbonne Universités, UPMC Univ Paris 06, Observatoire Océanographique, 06230 Villefranche-sur-mer, France; Department of Molecular and Cell Biology, University of California, Berkeley, CA 94720-3200 USA; Department of Biology, University of Miami, 208 Cox Science Center, 1301 Memorial Drive, Coral Gables, FL 33124 USA; Department of Biophysics, Graduate School of Science, Kyoto University, Kyoto, 606-8502 Japan; UMR 7144, CNRS and Sorbonne Universités Université Pierre et Marie Curie (UPMC) Paris 06, Station Biologique de Roscoff, Place Georges Teissier, 29680 Roscoff, France

**Keywords:** Choanocyte, Ephydatia, Microvilli, Porifera, Sponge, Stereocilia, Transcriptome

## Abstract

**Background:**

The microvillus is a versatile organelle that serves important functions in disparate animal cell types. However, from a molecular perspective, the microvillus has been well studied in only a few, predominantly vertebrate, contexts. Little is known about how differences in microvillar structure contribute to differences in function, and how these differences evolved. We sequenced the transcriptome of the freshwater sponge, *Ephydatia muelleri*, and examined the expression of vertebrate microvillar gene homologs in choanocytes—the only microvilli-bearing cell type present in sponges. Sponges offer a distant phylogenetic comparison with vertebrates, and choanocytes are central to discussions about early animal evolution due to their similarity with choanoflagellates, the single-celled sister lineage of modern animals.

**Results:**

We found that, from a genomic perspective, sponges have conserved homologs of most vertebrate microvillar genes, most of which are expressed in choanocytes, and many of which exhibit choanocyte-specific or choanocyte-enriched expression. Possible exceptions include the cadherins that form intermicrovillar links in the enterocyte brush border and hair cell stereocilia of vertebrates and cnidarians. No obvious orthologs of these proteins were detected in sponges, but at least four candidate cadherins were identified as choanocyte-enriched and might serve this function. In contrast to the evidence for conserved microvillar structure in sponges and vertebrates, we found that choanoflagellates and ctenophores lack homologs of many fundamental microvillar genes, suggesting that microvillar structure may diverge significantly in these lineages, warranting further study.

**Conclusions:**

The available evidence suggests that microvilli evolved early in the prehistory of modern animals and have been repurposed to serve myriad functions in different cellular contexts. Detailed understanding of the sequence by which different microvilli-bearing cell/tissue types diversified will require further study of microvillar composition and development in disparate cell types and lineages. Of particular interest are the microvilli of choanoflagellates, ctenophores, and sponges, which collectively bracket the earliest events in animal evolution.

**Electronic supplementary material:**

The online version of this article (doi:10.1186/s13227-016-0050-x) contains supplementary material, which is available to authorized users.

## Background

**Fig. 1:**
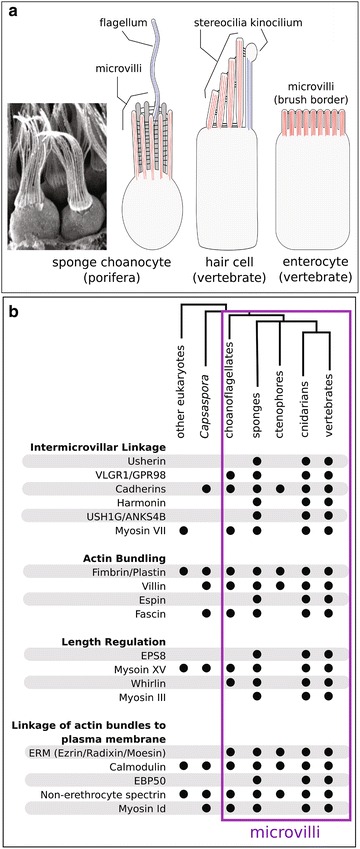
Phylogenetic distribution of microvilli and microvilli-associated proteins. **a** Sponge choanocytes have an apical ring of actin-cored (*red*) microvilli that are connected by intermicrovillar links. This structure called a “collar” surrounds a microtubule-cored (*blue*) flagellum that functions to generate flow through the water-canal system. Microvilli are found in diverse animal cell types. Two of the best-studied examples include mechanosensory hair cells of the vertebrate inner ear, and enterocytes of the vertebrate intestinal epithelium. **b** Phylogenetic distribution of microvillar proteins that are conserved between sponges and vertebrates

Specialized feeding cells of sponges, called choanocytes (Fig. 1), are central to discussions about animal cell type evolution due to their similarities with choanoflagellates, the unicellular/colonial sister group of animals [1–5]. Both cell types (generally described as “collar cells”) have an apical ring of actin-cored microvilli that surround a microtubule-cored flagellum—features that both lineages use for feeding on bacteria.

Whereas flagella/cilia are present in diverse eukaryotes and are very ancient [6], microvilli are unique to choanoflagellates and animals [7] and seem to represent an important innovation that has been co-opted for disparate functions in myriad animal cell/tissue types. In addition to bacterivory in choanocytes and choanoflagellates, microvillar functions include sperm recognition on oocytes, photoreception, chemosensation, bacterial defense and nutrient absorption in the intestine, and mechanosensation [8, 9]. In general, microvilli exhibit remarkable evolutionary versatility, yet little is known about their development, structure or function in any but a few cell types. Open questions remain about how differences in their molecular composition contribute to differences in their function, about how variations in their structure and organization (i.e., length, number) are regulated and, from an evolutionary perspective, how different cell types with microvilli are related—did they evolve through a vertical sequence of descent with modification, or can the microvillus be deployed de novo through activation of a conserved regulatory switch in otherwise unrelated cell types?

Perhaps the two best-studied examples of microvillar structure and function are enterocytes of the intestinal epithelium [10] and mechanosensory hair cells [11, 12] (Fig. 1). Enterocytes are adorned with hundreds of short, densely packed microvilli that comprise an organelle called the brush border. The brush border functions in nutrient absorption and defense against pathogens and toxins in the intestinal lumen [9]. In contrast vertebrate hair cells are found in both the ear and along the lateral line in fishes, and in both contexts function as sensory cells that use modified microvilli called stereocilia to transform mechanical stimuli (such as movement, sound vibrations or flow) into an electrical signal [12]. Stereocilia develop from shorter, more typical microvilli, but mature to have stair-stepped length gradations and a tapered base [9, 11].

Studies of the enterocyte brush border, hair cells, and—to a lesser extent—other vertebrate microvilli, such as in the retinal pigment epithelium [13], have identified a core set of proteins that (1) link neighboring microvilli to each other, (2) bundle and regulate actin dynamics in the microvillar core, (3) link the actin core to the microvillar membrane, and (4) regulate microvillar length [9]. Figure 1b illustrates the extensive conservation of these proteins between sponges and vertebrates, raising the possibility that these proteins also contribute the microvillar-collar structure of sponge choanocytes. Fewer of these proteins are conserved in choanoflagellates, and many are absent in ctenophores, which have microvilli on both oocytes [14] and putative sensory cells in the adult [15].

In general, there are too few data from non-vertebrate animals (and particularly non-bilaterian animals) to formulate clear hypotheses about the evolutionary sequence of origination and diversification of microvilli-bearing cell types. In this study, we examine microvillar gene expression in the choanocyte cells of two species of the freshwater sponge, *Ephydatia muelleri and Ephydatia fluviatilis*. Our results show that vertebrate microvillar genes are conserved and expressed in sponge choanocytes. These data suggest the deep evolutionary conservation of microvillar structure in animals and provide a foundation for comparative study of disparate microvilli-bearing cell types in animals and choanoflagellates.

## Results

### *E. muelleri* reference transcriptome

Assembly of the *E. muelleri* transcriptome resulted in 85,971 transcripts encoding 29,154 predicted peptides of >50aa in length. Assembled transcripts and predicted peptides are available at compagen.org [16].

### Hydroxyurea treatment to inhibit choanocyte differentiation

In *Ephydatia*, each individual produces thousands of gemmules, which are resistant spore-like structures that contain hundreds or more genetically clonal thesocytes (resting stem cells that contain extensive nutrient reserves) [17]. Gemmules can survive unfavorable conditions that kill the adult sponge, and when favorable conditions return, gemmules undergo germination—a process in which thesocytes divide within the gemmule to produce archeocytes (adult stem cells), which then exit the gemmule and differentiate to re-form adult tissues [18]. Germination occurs within 72 h of placing a gemmule at room temperature, and within 90–120 h, choanocytes begin to differentiation from archeocytes [19, 20].

**Fig. 2:**
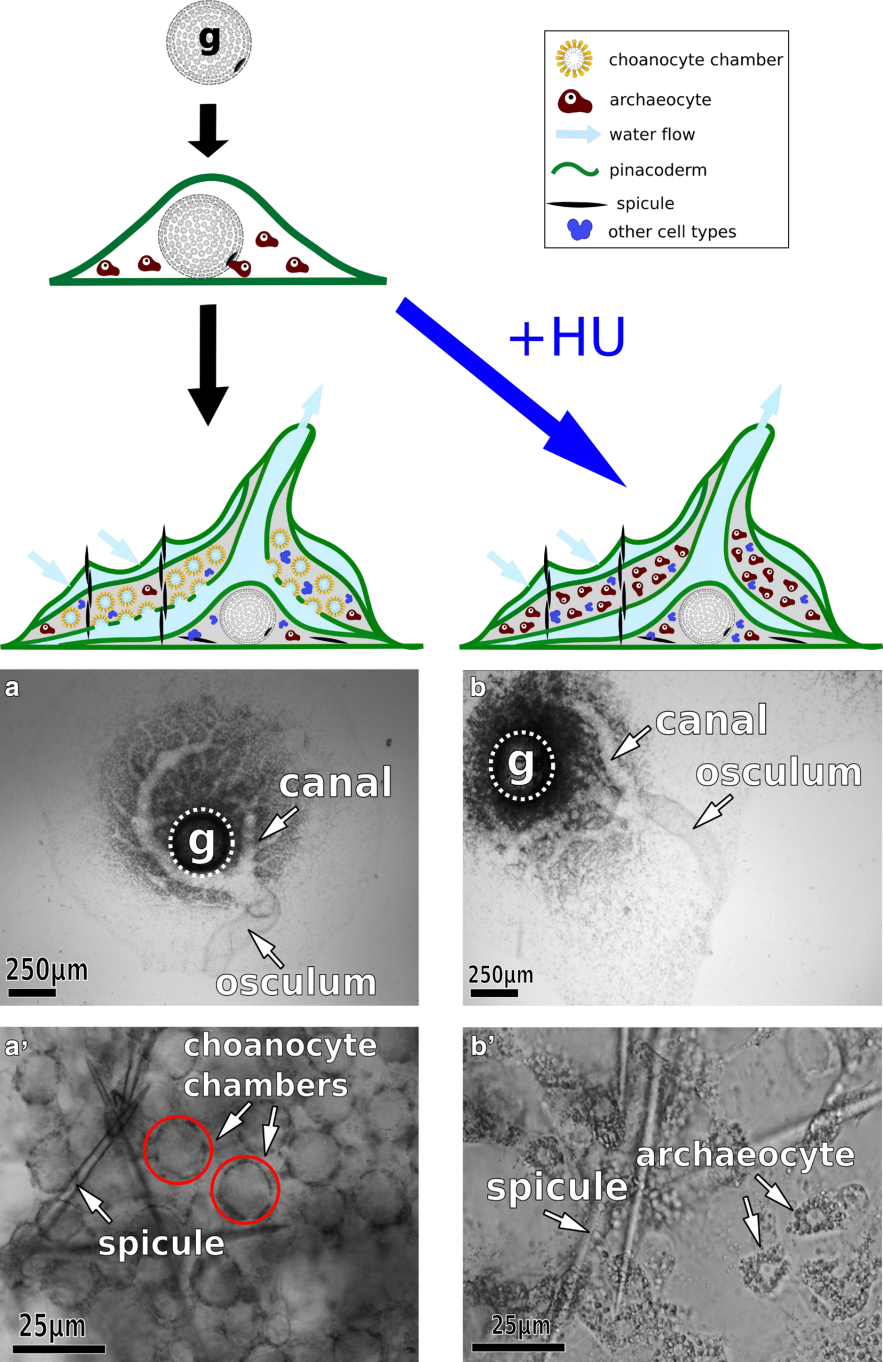
Hydroxyurea inhibits choanocyte differentiation. Addition of hydroxyurea (HU) during gemmule hatching, just prior to the differentiation of choanocytes, leads to sponges that lack choanocytes and are enriched for archeocytes, their developmental precursors. **a**/**a**’ Low- and high-magnification images a no-treatment control sponge, whereas **b**/**b**’ show comparable views of an HU-treated sponge (*g* gemmule)

In a previous study, Rozenfeld and Razmont [19] applied hydroxyurea (HU)—a DNA synthesis inhibitor—to gemmules throughout germination and reported widespread effects on development, including the lack of choanocytes, a canal system, and an osculum. If HU was applied 90–120 h post-germination (i.e., the point at which all juvenile tissues had begun to differentiate), no developmental defects were detected and choanocyte chambers fully formed. We modified this approach to apply HU just prior to choanocyte differentiation (see “Methods” for details). We found that this approach minimized developmental defects and that HU-treated sponges developed all observable features of the untreated control sponges, except that they lacked choanocytes. Specifically, archeocytes, water canals, an osculum, and spicules (and therefore sclerocytes—the cells that produce spicules) were detected in both control and HU-treated sponges (Fig. 2). Unobserved effects on cell types other than choanocytes are probable, but the predominant difference between control and HU-treated sponges was the presence versus absence of choanocytes, respectively.

### Differential gene expression analysis in *E. muelleri*

The premise of our approach was that the transcripts which show some degree of choanocyte-specific or choanocyte-enriched expression under normal conditions should exhibit lower expression levels in HU-treated sponges that lack choanocytes. Between 21.1 and 29.3 million reads were sequenced from each of six biological replicates (three control samples and three HU-treated samples). Reads were mapped to clusters using Corset, and transcript diversity was further reduced to 30,033 (from 85,971) using EdgeR to remove clusters with fewer than 1 counts per million (cpm) in at least three biological replicates (recommended by the EdgeR manual). Of these, 2940 clusters showed at least twofold (log FC < −1) lower expression in HU-treated samples than in untreated control samples, with a false discovery rate (FDR)-corrected *p* < 0.05. In comparison, analysis of these same data using Kallisto/Sleuth, and applying the same significance criteria, resulted in 1062 transcripts with significantly lower expression levels in HU-treated samples than in untreated control samples. Kallisto differs from read counting methods in that it pseudoaligns reads to a set of transcripts without assigning each read to a specific set of coordinates within the transcript. Furthermore, the speed of Kallisto allows for the use of bootstrapping to test the uncertainty in transcript abundance estimates, which can stem from high similarity among transcript isoforms [21]. Sleuth is a companion program that uses Kallisto results, including an error model incorporating Kallisto’s bootstrapping, to differentiate between true biological expression differences and variation resulting from sources of experimental noise [22].

### Microvillar gene expression in *Ephydatia* choanocytes

**Fig. 3:**
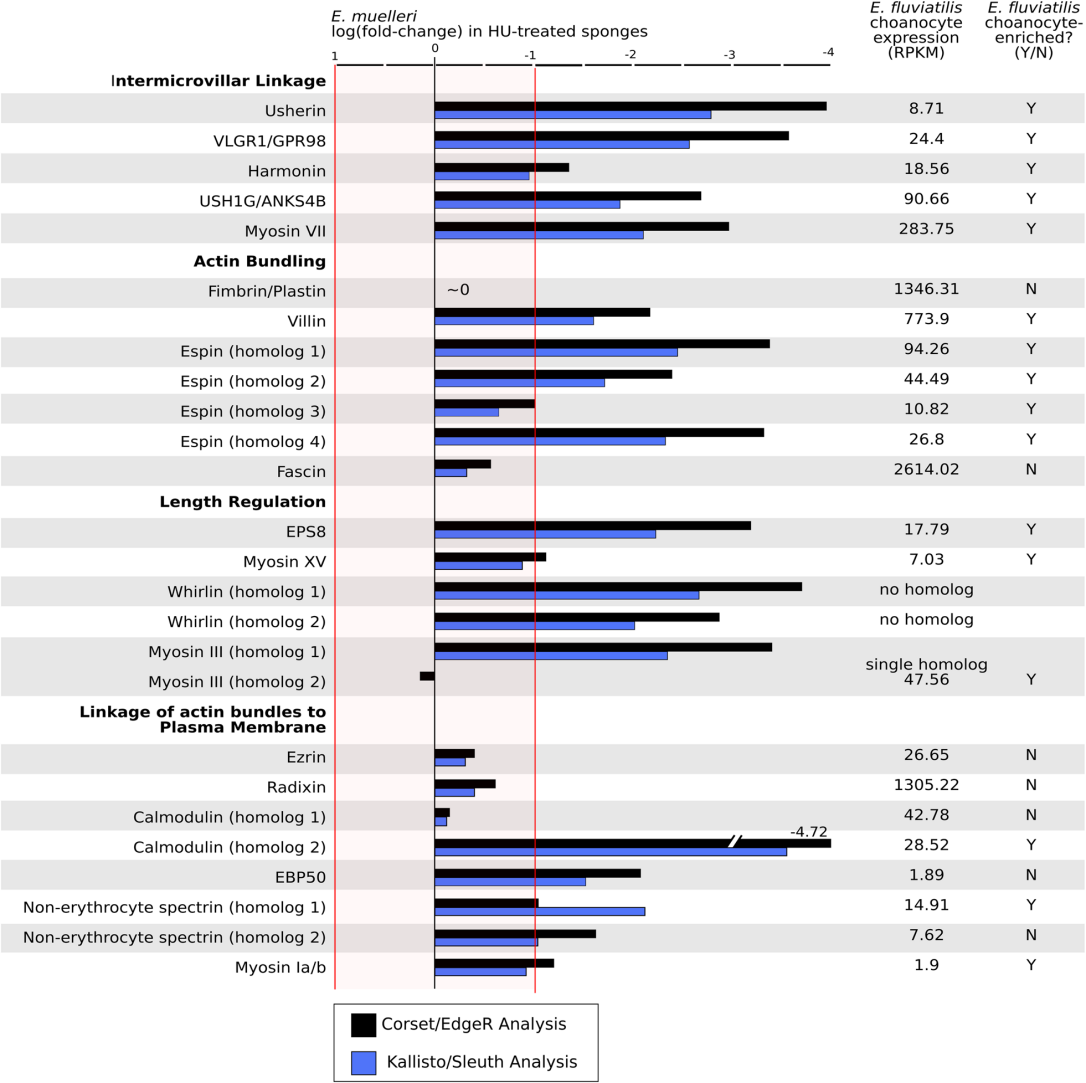
Choanocyte expression of vertebrate microvillar gene homologs. In *E. muelleri*, choanocyte expression of candidate microvillar genes was examined through differential expression analysis of HU-treated (i.e., choanocyte-absent) sponges relative to untreated (i.e., choanocyte-present), control sponges. Genes with significantly lower expression in HU-treated sponges (log FC < −1) are interpreted as having elevated expression levels in choanocytes of normal, untreated sponges. In contrast, in *E. fluviatilis*, choanocyte gene expression levels (RPKM) were determined by direct sequencing of isolated choanocytes and were compared to choanocyte-free cell fractions (summary of expression values is provided in Additional file 2: Supplemental Table 1)

**Fig. 4:**
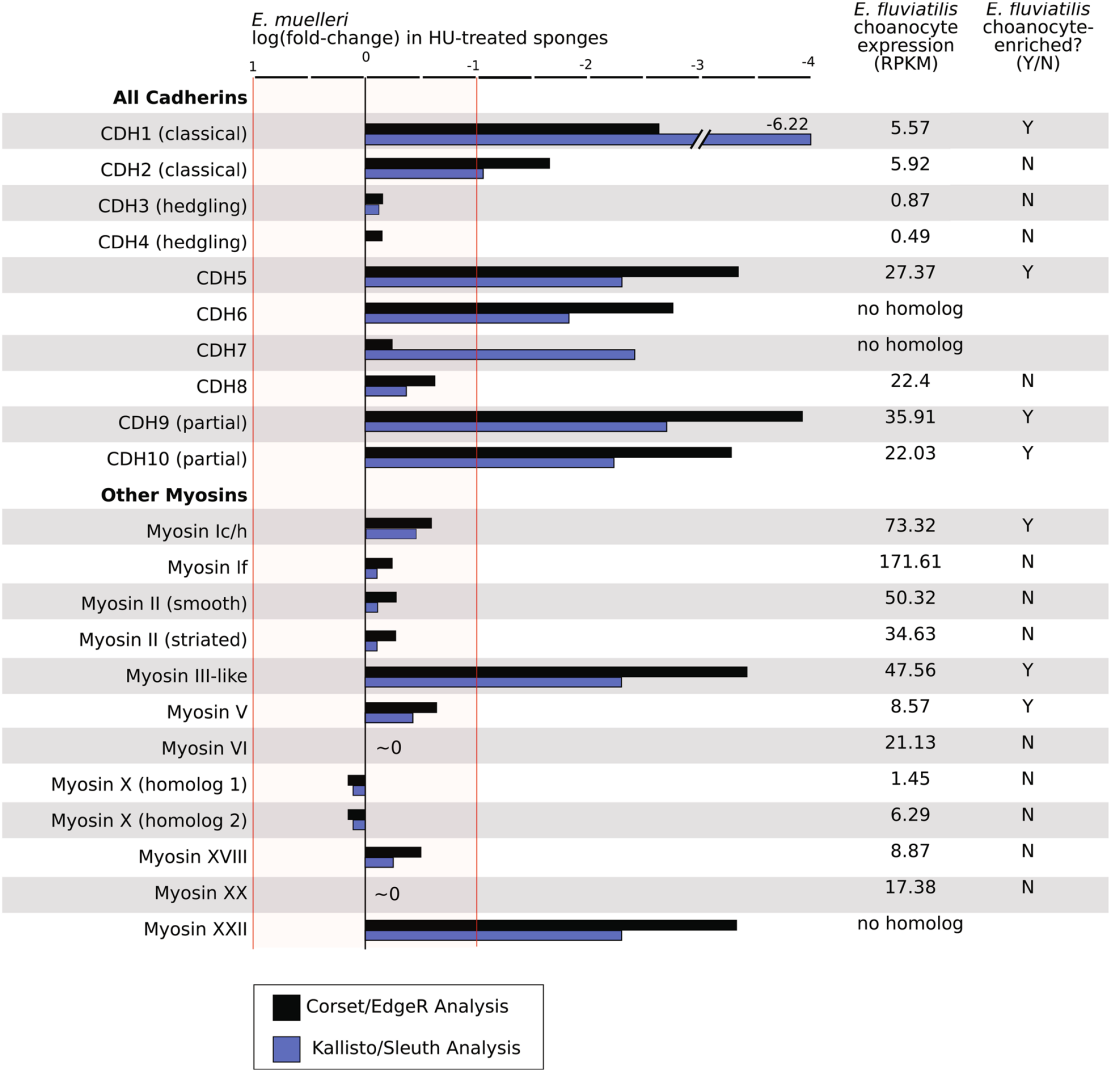
Choanocyte expression of cadherins and myosins. Diverse cadherins and myosins regulate the development, structure, and function of microvilli on disparate cell types. We examined choanocyte expression of the full catalog of cadherins and mysoins detected in the *E. muelleri* transcriptome, and their orthologs identified in the *E. fluviatilis* transcriptome (summary of expression values is provided in Additional file 2: Supplemental Table 1)

To test for choanocyte expression of conserved microvillar genes, we searched for sponge homologs of genes known to regulate the development, structure, and function of microvilli in other animals—predominantly vertebrate hair cells and enterocytes. Highly conserved homologs of most of these genes were identified by best reciprocal BLAST search against the *Ephydatia* transcriptome, and positive hits were validated by examining their domain organization, and in some cases through phylogenetic analysis of putative sponge homologs (e.g., myosins; Additional file 1: Supplement Figure 1). All identified *E. muelleri* homologs of vertebrate microvillar genes are listed in Fig. 3. We examined choanocyte expression of the full complement of *Ephydatia* cadherins and myosins (Fig. 4) because different microvilli often express different members of these protein families, and because cadherin orthology cannot be reliably determined between vertebrates and sponges [23]. The only vertebrate microvillar genes that were not detected in the *Ephydatia* transcriptome were Prominin [24], Xirp2 [25], Stereocilin [26], Fasciclin 2 [27], Cordon Bleu [28], mucosal barrier-associated mucins [9], and specific intermicrovillar linker cadherins (discussed below).

Choanocyte gene expression was previously characterized in *Ephydatia fluviatilis*, which is very closely related to *E. muelleri*, and gene orthologs were identified between these two species using nucleotide BLAST searches. No *E. fluviatilis* ortholog was detected for the *E. muelleri* homologs of Whirlin, CDH6, CDH7, or Myosin XXII. Furthermore, the *E. fluviatilis* transcriptome assembly encodes a single Myosin III homolog, whereas two were detected in *E. muelleri*.

### Intermicrovillar linker proteins

Despite the stark structural and functional differences between the enterocyte brush border and hair cell stereocilia, it is now apparent that their structure is regulated by a similar protein complex that includes two cadherins that function in intermicrovillar adhesion, two scaffolding factors, and a myosin motor protein. In the brush border, this complex is called the intermicrovillar adhesion complex (IMAC) and in hair cells is called the Usher complex (because its disruption contributes the hearing loss associated with Usher syndrome) [29, 30]. With the exception of Harmonin, a PDZ-containing protein which is the primary scaffolding protein in the brush border and stereocilia, the IMAC and Usher complex are otherwise composed of different paralogs of the same protein families, suggesting that these cell types diverged from a common ancestral cell type, and that their divergence was coincident with duplication and divergence of IMAC- and Usher complex-related genes.

The most highly conserved components of the IMAC/Usher complex are Harmonin, USH1G/ANKS4B, and Myosin VII [29, 30]. The *E. muelleri* transcripts encoding homologs of these proteins showed twofold–fourfold lower expression in HU-treated sponges than in controls and were detected as expressed and enriched in choanocytes of *E. fluviatilis* (Fig. 3).

The specific cadherins that form intermicrovillar links in the enterocyte brush border include PCDH24 and MLPCDH [31]. In contrast, Cadherin 23 and Protocadherin 15 form tip-links between adjacent stereocilia in vertebrate hair cells [32]. A putative Cadherin 23 homolog has also been detected at tip-links that connect mechanosensory stereocilia in the cnidarian, *Nematostella vectensis* [33, 34]. We detected 10 candidate cadherin transcripts in *E. muelleri* transcriptome. Two encode classical cadherins, which have well-characterized cell–cell adhesion functions in bilaterians [35], and two encode hedgling homologs, which have putative developmental signaling functions (Fig. 4) [36]. The six remaining cadherins have no obvious orthology to known microvillar cadherins. We found that both classical cadherin homologs had relatively low, but similar expression levels in *E. fluviatilis* choanocytes, but that one was significantly enriched in *E. fluviatilis* choanocytes and showed greater than 12-fold lower expression in HU-treated *E. muelleri* samples than in controls. It is possible that this classical cadherin functions in the microvillus, but based upon our knowledge of classical cadherin function in bilaterians, it is more likely to be involved in cell–cell adhesion within the choanoderm.

**Fig. 5:**
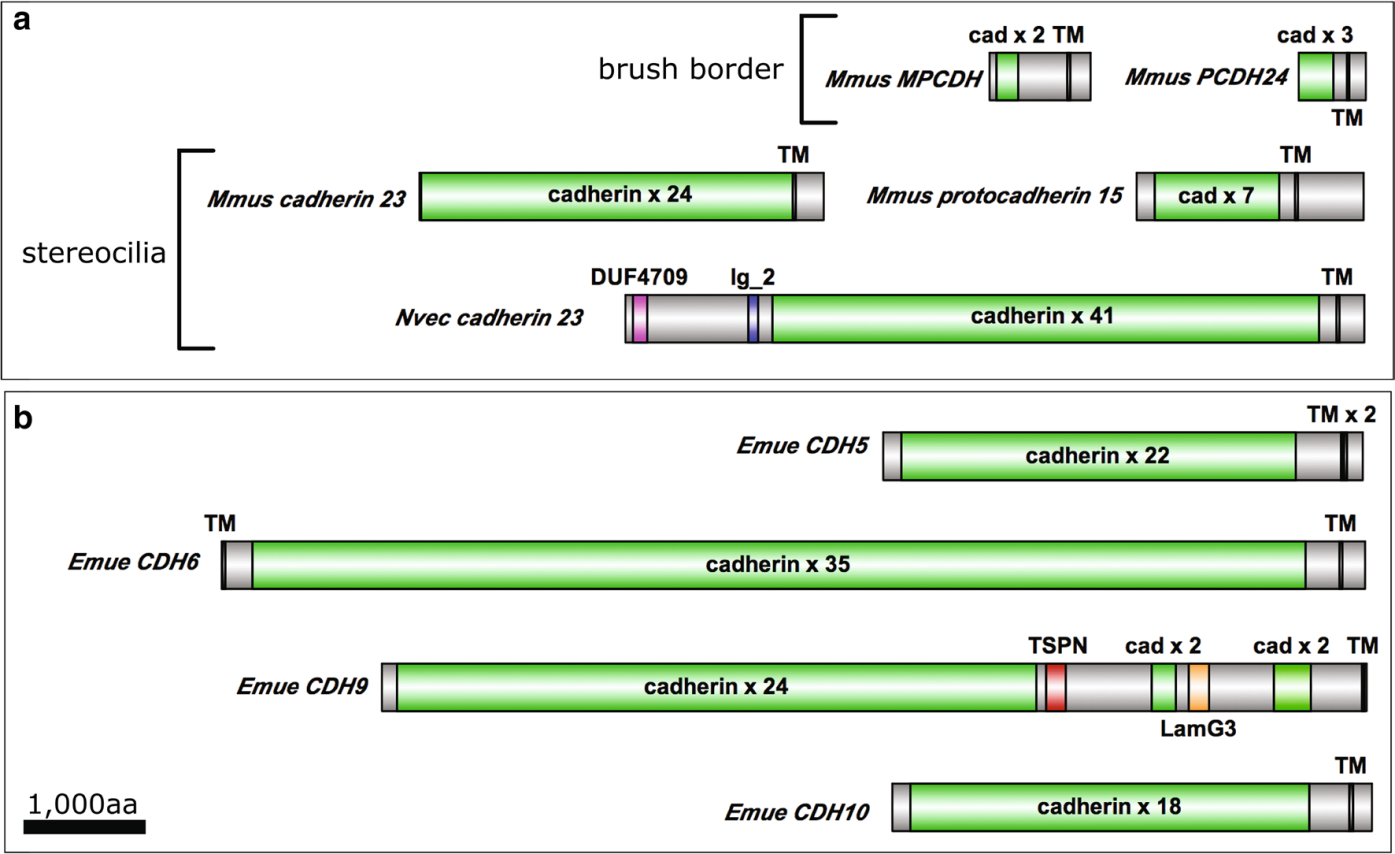
Domain architecture of known and candidate intermicrovillar-link cadherins. **a** Cadherins that link microvilli of the enterocyte brush border, and stereocilia of hair cells of vertebrates, and hair bundles of the cnidarian *N. vectensis*. **b** Four cadherins are predicted to have elevated expression levels in choanocytes of *E. muelleri*. None have obvious orthology with known microvillar-link-forming cadherins, but may function in this capacity (Mmus = *Mus musculus*, Nvec = *Nematostella vectensis*, Emue = *Ephydatia muelleri*, cad = cadherin, TM = transmembrane, DUF = domain of unknown function, TSPN = tetraspanin, LamG3 = Laminin G3)

Four uncharacterized cadherins also showed twofold–eightfold lower expression in HU-treated *E. muelleri* samples than in untreated controls. Three of these had detectable orthologs in the *E. fluviatilis* transcriptome, and all were expressed and enriched in choanocytes. The domain architecture of these cadherins provided few clues about their possible affinity with vertebrate or cnidarian cadherins involved in intermicrovillar linkage (Fig. 5).

In addition to tip-links, hair cell stereocilia also exhibit lateral links and ankle links. Two proteins that contribute to these structures are Usherin and VLGR1/GPR98 [37–39]. Both are required for normal stereocilia development, and mutations in each are associated with Usher syndrome. We found that both have conserved orthologs in sponges, and VLGR1 is even present in choanoflagellates (Fig. 1). Moreover, both showed fivefold–eightfold lower expression in HU-treated sponges than in controls and were detected as expressed and enriched in the choanocytes of *E. fluviatilis* (Fig. 3).

### Actin-bundling proteins of the microvillar core

Microvilli and stereocilia are supported by bundles of polarized actin filaments with the plus-ends pointing toward the tips. Actin cross-linking proteins shown to be associated with the microfilament core include Fimbrin (also known as Plastin), Villin, Espin, and Fascin [7, 9]. Villin and Espin are both implicated in microvillar length regulation in addition to microfilament bundling/cross-linking.

The identity and abundance of different actin-bundling proteins varies between microvilli in different contexts. For example, Villin and Fimbrin are the two major actin-bundling proteins in the enterocyte brush border, whereas stereocilia lack Villin altogether and instead contain Fimbrin and higher levels of Espin. Villin is also reported as absent in placental microvilli and studies of the mouse retinal pigment epithelium have failed to detect both Villin and Fimbrin as microvillar components (reviewed in [9]).

We found that the most highly expressed actin-bundling proteins expressed in choanocytes of *E. fluviatilis* are Fascin, Fimbrin, and Villin, with relatively low expression levels detected for four Espin homologs (Fig. 3). However, only Villin and Espin were significantly enriched in choanocytes of both *Ephydatia* species. Fascin is not commonly reported as a component of microvilli in normal vertebrate tissues, but has been detected in the microvilli of human glioma cells and in the choanoflagellate, *Salpingoeca rosetta*.

### Proteins involved in microvillar length regulation

Microvillar length is tightly regulated. In both sponge choanocytes and the enterocyte brush border, microvilli on each cell are of uniform length, whereas stereocilia of cnidarian hair bundles and vertebrate hair cells have stair-stepped length gradations related to their function as mechanosensors. However, it is difficult to distinguish between proteins that affect microvillar length and those that regulate it. For example, experimental knockdown of the actin-bundling proteins discussed above each results in shorter microvilli [40], but this may be a simple biophysical result of reduced stiffness of the actin core [9].

In the stereocilia of mouse hair cells, it has been shown that length regulation by Myosin XV and Whirlin is dependent on the actin-capping protein EPS8. Disruption of any of these components reduces stereociliary length. All three are components of the tip complex, where the actin-capping activity of EPS8 is an important regulatory element for elongation of the actin core [41]. We found that *E. muelleri* has one EPS8, one Myosin XV, and two Whirlin homologs, and that EPS8 and both Whirlin homologs showed fivefold–sevenfold lower expression in HU-treated sponges than in controls. We also found that Myosin XV was significantly enriched in Corset/EdgeR analyses, but was below the significance threshold in Kallisto/Sleuth analyses. Both EPS8 and Myosin XV were expressed and enriched in choanocytes of *E. fluviatilis*, but no Whirlin ortholog was detected in the transcriptome of this species.

Two additional proteins that regulate microvillar length are Myosin III and Cordon Bleu. Myosin III constricts stereociliary tips in hair cells and may downregulate the function or limit the access of actin-binding/regulatory proteins to microfilament ends [42]. Cordon Bleu regulates microvillar length in placental syncytiotrophoblasts in a manner that is not completely understood, but may be dependent upon the actin severing activity of its WH2 domains [28]. We found that one of two Myosin III homologs showed fivefold–sevenfold lower expression in HU-treated *E. muelleri* samples, and that its ortholog in *E. fluviatilis* was choanocyte-enriched. In contrast, neither species contains a Cordon Bleu homolog.

### Proteins that tether the actin core to the microvillar membrane

In the brush border, the most abundant myosin is Myosin Ia, which interacts with calmodulin to link the bundled actin core to the microvillar membrane [43]. Of two detected calmodulin homologs in *Ephydatia*, we found that both were expressed in choanocytes of *E. fluviatilis*, but only one was enriched. The *E. muelleri* ortholog of this gene also showed >ninefold lower expression in HU-treated samples than in the controls. Both species have a single Myosin in the Ia/b family (Additional file 1: Supplement Figure 1), and despite low overall expression (RPKM = 1.9), it was supported as choanocyte-enriched in *E. fluviatilis* and was near the significance threshold in *E. muelleri*.

Unlike the condition in the brush border, in *E. fluviatilis* Myosin Id was detected as the most highly expressed myosin homolog in choanocytes, and we found it to be enriched in choanocytes of both species (Fig. 4). This is interesting because previous studies have shown that Myosin Ia depletion leads to an increase in microvillar concentrations of Myosin 1d [44], suggesting that in the intestinal brush border, Myosin Id can substitute for Myosin Ia.

Another important class of proteins that function in membrane linkage (in addition to important regulatory functions) in the microvillus is the ERM family. The ERM family is composed of three closely related paralogs, Ezrin, Radixin, and Moesin. Their functions in microvilli have been reviewed in detail elsewhere [9], but it is worth noting that they exhibit interesting patterns of tissue-specific localization. Ezrin is enriched in microvilli of many epithelial tissues, whereas Moesin is enriched in endothelial microvilli [45], and Radixin is predominantly found in hair cell stereocilia [9]. These proteins interact with PIP2 in the inner leaflet of the microvillar membrane and with the scaffolding protein EBP50 to create a bridge between the microfilament core and the membrane [9, 46, 47].

We found that *Ephydatia* has homologs of Ezrin and Radixin, but not Moesin (Fig. 3). Both are expressed in choanocytes of *E. fluviatilis*, but neither are choanocyte-enriched in either species of *Ephydatia*. However, similar to hair cell stereocilia, Radixin had >50-fold higher expression in choanocytes than did Ezrin.

Finally, actin filaments at the base of microvilli are stabilized by a “terminal web” that contains the membrane-associated scaffolding protein, non-erythrocyte spectrin [48]. We identified two homologs of this protein in *Ephydatia* and found that both showed significantly lower expression in HU-treated *E. muelleri* samples, both were expressed in *E. fluviatilis* choanocytes, and that one was enriched in *E. fluviatilis* choanocytes.

## Discussion

The ubiquity and functional diversity of microvilli [8] in animals and choanoflagellates raises challenging questions: How does the molecular composition of microvilli in different cell types vary, and how does this variation contribute to structural and functional differences? Through what sequence did microvilli-bearing cell types diversify? Which microvillar functions are ancient/derived?

The answers to these questions depend upon detailed study of the molecular composition of microvilli in diverse animal cell and tissue types. To date, these kinds of data are available for only a handful of, predominantly vertebrate, cell types. In this study, we examined the expression of vertebrate microvillar gene homologs in choanocytes—the only microvilli-bearing cell type of sponges. In addition to sponges being an evolutionary outlier for distant phylogenetic comparison with vertebrates, choanocytes are of special interest because of their long-hypothesized similarity to choanoflagellates. Either choanocytes or choanoflagellates utilize their microvillar collar for bacteriverous feeding because this is the ancestral condition, from which microvilli in all animals are derived, or they have independently co-opted the microvillus for this function. Either way, our understanding of the earliest events in microvillar evolution depends upon the comparative study of choanoflagellates, sponge choanocytes, and the various other microvillar-bearing cell types present in non-bilaterian animal lineages.

As a first step toward understanding the molecular composition of choanocyte microvilli, we sequenced and assembled the transcriptome of the freshwater demosponge *Ephydatia muelleri.* This transcriptome was used as a reference to detect changes in global transcript abundance in response to drug treatments that blocked choanocyte differentiation during development from gemmules. Genes with lower expression in HU-treated sponges (i.e., sponges without choanocytes) were interpreted as normally having choanocyte-specific or choanocyte-enriched expression. The efficacy of this approach is strongly supported by nearly perfect corroboration of *E. muelleri* results by those independently obtained from isolated choanocytes in the related species, *E. fluviatilis* [49].

The main finding of this study is that many of the core microvillar proteins known from well-studied systems—principally vertebrate enterocytes and hair cells—are evolutionarily conserved in sponges and most exhibit elevated expression levels in choanocytes. We examined four categories of proteins: (1) intermicrovillar linker proteins, (2) actin-binding proteins of the microvillar core, (3) proteins that regulate microvillar length, (4) proteins that tether the actin core to the microvillar membrane. To the extent that the expression of microvillar gene homologs in choanocytes is a proxy for their involvement in the microvillus, our data present a portrait of extensive conservation between sponge and vertebrate microvilli.

From these data we would hypothesize that the identified microvillar links of the sponge collar are composed of one or more of the four identified choanocyte-expressed cadherins, which presumably interact with harmonin, ANKS4B, and Myosin VII [29, 30]. These genes are all choanocyte-enriched, and this microvillar linker complex is conserved in enterocytes and hair cells. Choanocytes also express Usherin and VLGR1 which form links between hair cell stereocilia but not between microvilli of the enterocyte brush border. This highlights that despite the conservation of microvillar proteins overall, specific microvillar components and their abundance may vary significantly between cell types. Other examples include the actin-bundling proteins Fimbrin and Villin—both are highly abundant in the brush border and highly expressed in choanocytes, but Villin is entirely absent in hair cell stereocilia. Likewise, ERM family proteins show tissue-specific involvement in microvilli, with Ezrin as the predominant component of the brush border, and Radixin as the predominant component of stereocilia (reviewed in [9]). In choanocytes of *E. fluviatilis*, Radixin showed 50-fold higher expression than Ezrin, although neither was choanocyte-enriched.

Overall, our study suggests that the molecular composition of microvilli in sponge choanocytes falls within the range of divergence exhibited between microvilli in different vertebrate cell types. However, the evolutionary implications of this finding are less clear, principally because choanoflagellates and ctenophores also have microvilli but both lineages seem to lack homologs of fundamental microvillar genes (Fig. 1). This suggests that, at the molecular level, microvilli in these lineages are considerably different from microvilli in sponges and vertebrates. This may reflect the ancestral condition, a derived condition, or perhaps more likely—a combination of both, and warrants further investigation.

A limitation of this study is the reliance on gene expression data. In the future it would be preferable to conduct proteomic analyses on directly isolated choanocyte microvilli. A possible strategy to achieve this may be the use of lectin-decorated agarose beads that interact with microvillar-associated glycoproteins; this technique has proven effective to isolate microvilli from the retinal pigment epithelium in mice [13]. Not only would this approach provide more direct evidence for microvillar localization of candidate microvillar proteins, but would enable the comprehensive discovery of sponge microvillar components rather than using a candidate gene approach based upon prior knowledge from vertebrates.

## Conclusions

We have shown that the core molecular components of the enterocyte brush border and hair cell stereocilia of vertebrates are conserved in sponges and that the majority exhibit choanocyte-specific/enriched expression. These data are consistent with the view that the microvillus is a versatile organelle that, once evolved, was repurposed for myriad functions in disparate animal cell types.

## Methods

### Live materials

*Ephydatia muelleri* gemmules were collected from Red Rock Lake, Colorado, USA (Em-CO); Beavertail Lake, Vancouver Island, Canada (Em-BTL); and Nanaimo River, Vancouver Island, Canada (Em-NR). The gemmules were stored in autoclaved lake water, in the dark at 4 °C.

### Reference transcriptome

#### Quality trimming

We performed read trimming using Trimmomatic version 0.30 [50] and implemented two separate filters: (1) removal of TruSeq adapter sequence and (2) trimming low-quality bases from the ends of each read. To accomplish this, we ran Trimmomatic in three phases. In the first phase, we clipped palindromic adapters using the directive ILLUMINACLIP:2:40:15 and we discarded resulting reads fewer than 25 bases with MINLEN:25. This resulted in two data sets: one set with reads whose mate pair remained in the set, and the other composed of forward reads whose reverse pair was removed due to adapter contamination (no reverse unpaired reads remained, as they would have been removed by the adapter clipping). The second phase operated on the remaining paired data set. We clipped simple adapters using the directive ILLUMINACLIP:2:40:15, and we imposed a minimum Phred-like quality cutoff of 5 on the first ten and last ten bases using LEADING:5 and TRAILING:5, subjected the read to a minimum sliding window quality using SLIDINGWINDOW:8:5 and discarded resulting reads shorter than 25 bases with MINLEN:25. We used a permissive minimum Phred-like quality of 5 only in order to remove obviously noisy bases, as these might interfere with read error correction in the subsequent step of our processing. The third phase operated on the unpaired forward reads from the first phase and implemented the same directives as the second phase. We chose a minimum read length of 25 because that is the k-mer length for the de novo assembly program we used, Trinity, and so reads shorter than 25 bases would not be included in assemblies. In all adapter clipping operations, we used adapter sequences appropriate to the index used for multiplexed sequencing. When clipping palindromic adapters, we used as input the sequence of the TruSeq Universal Adapter and the reverse complement of the index-specific adapter. When clipping simple adapters, we used as input the sequence of both universal and specific adapters and their reverse complements.

#### Error correction

We conducted read error correction on trimmed reads using Reptile v1.1 [51] following the steps described by the authors, with modifications described below. We began by using the “fastq-converter.pl” script to convert from FASTQ and to discard reads with more than 1 ambiguous character (N) in any window of 13 bases. We chose the character “a” as the target to convert ambiguous bases in reads surviving this filter, as all of the characters in our input reads were in upper case (A, C, G, or T); thus, we could later recognize ambiguous bases converted in this step. Next, we tuned parameters using the “seq-analy” utility included in the software package, again following the instructions provided by the authors, in four steps: (1) Running “seq-analy” with default settings. (2) Adjusting the input settings to “seq-analy” using the results of the first run. In our case, we set QThreshold to 73, MaxBadQPerKmer to 8, and KmerLen to 25 (to match the k-mer length used in Trinity). (3) Rerunning “seq-analy” using the adjusted input settings. (4) Creating the input settings to Reptile based on the output of step 3. In our case, we set T_expGoodCnt to 44, T_card to 17, KmerLen to 13, Qthreshold to 73, Qlb to 60, MaxBadQPerKmer to 8 and Step to 12, leaving all other settings at their defaults. Using these settings, we next ran Reptile to identify errors in our trimmed reads.

In examining the errors that were identified by Reptile, we noticed that they fell into two classes regarding their locations within reads: sporadic errors not located adjacent to any other error that was identified, and clustered errors, in which several or even all adjacent bases within the same k-mer window were corrected. In some extreme cases, every single base within a sequence read was identified as a target for error correction. We reasoned that this was an unintended consequence of the iteration-to-exhaustion approach taken by Reptile (step 2d of the algorithm described in section 3.1 of the Reptile manuscript). Therefore, we designed a custom Perl script to correct sporadic errors but not clustered errors. We began by grouping each read according to the total number of errors identified within the read. For each group, we built a distribution of the number of other errors identified adjacent to each error within the same k-mer window. For sporadic errors, this number should be close to 0, but for clustered errors, the number could be up to the k-mer size minus one. There was a clear pattern within each of these distributions, with a number of errors identified with no neighbors, a smaller number identified with 1 neighbor, and an increasing number beginning at 2 or more neighbors. The first categories represent sporadic errors, and the increasing categories represent clustered errors. Therefore, we used these empirical distributions to set a cutoff for the maximum number of allowed neighbors within each group, by setting the maximum allowable amount of neighbor errors within a k-mer window to be the count just prior to the beginning of the secondary increase within each distribution. For example, in the case of the group of reads containing 4 total identified errors, there were 347,816 errors with no neighbors within the same k-mer, 163,548 with one neighbor, 262,814 with 2 neighbors, and 2549,510 with 3 neighbors (that is, all 4 errors were within the same k-mer window), and thus, our maximum allowed number of nearby errors was 1. After implementing Reptile’s suggested error correction of sequence reads and quality files subject to these cutoffs, we performed a final step of restoring ambiguous bases converted by “fastq-converter.pl” that were not corrected by Reptile back to their original value of “N.”

#### De novo transcriptome assembly

We performed de novo transcript assembly on the trimmed, corrected sequence reads and quality files with Trinity release 2013-02-25 [52] using “–min_kmer_cov” of 2 and “–SS-lib_type” to “RF” (for strand-specific reads), and all other parameters set to their defaults. This resulted in an initial assembly containing 148,449 contigs.

#### Identification and removal of cross-contamination

Cross-contamination within a sequencing lane is a known phenomenon, and it is estimated to cause incorrect assignment of roughly 0.5 % of index pairs within the same lane [53]. The transcriptome of *E. muelleri* was sequenced together in the same lane as an undescribed species of *Oscarella* (in preparation), so we designed a procedure to identify and eliminate cross-contaminated contigs present in the *de novo* assemblies. To identify putatively cross-contaminated contigs, we ran the blastn program from the BLAST package version 2.2.26 [54] with an expectation value of 1 × 10^−10^ to match contigs from each species against the other species. In order to separate a contaminating sequence from a truly homologous one, we were forced to make heuristic decisions in choosing cutoffs for two properties of the BLAST hits: percent match and match length. In examining the BLAST hits, we reasoned that perfect matches between two species should be the result of cross-contamination. We also reasoned that highly conserved genes would be nearly identical but not perfect matches between any two species. To determine whether we could separate these two categories of matches, we examined the percent match distribution of all cross-species BLAST hits. We found that the majority of putative cross-contaminated matches were perfect. However, sequencing errors are more likely to affect the final sequence of a cross-contaminated contig rather than a non-contaminated contig, as only a small number of reads should have found their way into the contaminated assembly. Therefore, percent identities slightly less than 100 % are expected for cross-contaminated contigs. We found that there appeared to be a separation between the number of BLAST hits at less than 96 % identity and the number at 96% or greater, and so we chose a minimum of 96 % identity for this cutoff. We explored match length in a similar manner and chose a minimum match length of 90 bases as our second cutoff.

Next, we identified the source of each putative contaminated contig. We reasoned that if cross-contamination occurred due to the misreading of a small number of index reads that produced a set of assembled cross-contaminated contigs, there should be a large discrepancy between the number of sequence reads mapping to the contigs that originated from the species that was the source of contamination versus the species that was contaminated. To test this hypothesis, we aligned all reads within a sequencing lane to all of the contigs produced within the lane. While implementing the alignment process, we noticed that there were a small number of contigs with ten thousand or more reads mapping from all species sequenced within the same lane. These contigs all had relatively short tandem repeats, and we observed that each of our sequence data sets also contained a relatively large number of reads (on the order of 10^5^) passing our quality trimming filters but also containing short tandem repeats. As these tandem repeats would interfere with our measurement of the source of contamination, we masked contigs with Tandem Repeats Finder version 4.04 [55], with the following parameter values: match = 2, mismatch = 7, indel penalty = 7, match probability = 80, mismatch probability = 10, min score = 30, max period = 24. All parameter values were taken from the default values on the Tandem Repeats Finder Web site, except for the minimum score to report and the maximum period of the tandem repeat. We chose 24 as the maximum period of the tandem repeat, as we wanted it to be smaller than the k-mer size of 25 used in Trinity, and we chose 30 as the minimum score to report, as this would be the score of a 24-base-long sequence containing tandem repeats with 2 bases not corresponding perfectly to the predicted repeat pattern (a score of 2 times 22 matches minus a score of 7 times 2 mismatches or indels).

We mapped reads to masked contigs using the Burroughs-Wheeler Aligner, BWA, version 0.7.5a [56], and we determined read mapping counts using SAMtools version 0.1.18 [57]. We ran BWA “aln” with the “-n 200” option to allow up to 200 equally best hits to be reported. All other BWA parameter values were set to their defaults. Using the read mappings, for each putatively cross-contaminated contig we identified using the BLAST strategy described above, we counted the number of reads mapping from the sponge species that produced the contig and compared it to the number of reads mapped from the other sponge species. To eliminate cross-contaminated contigs, we removed all contigs identified as putatively cross-contaminated that did not have at least 10 times as many reads mapping from the sponge species that produced the contig as from the other species, with one exception: If a contig had at least 10,000 reads mapping from the species that produced the contig, we did not discard it, regardless of read mapping ratio. We applied this exception because we observed that the most highly expressed transcripts (e.g., alpha tubulin and elongation factor 1 alpha) also tended to be the most conserved, and thus, the read mapping ratio was often close to 1 for these contigs. The decontamination process resulted in the removal of 581 contigs, leaving 147,868 contigs remaining.

#### Prediction of amino acid sequences from assembled transcripts and elimination of redundant transcripts

We used Transdecoder release 2012-08-15 [http://transdecoder.github.io/] to predict amino acid sequences from decontaminated assembled transcripts, with a minimum protein sequence length of 50, resulting in 101,671 predicted proteins. We noticed that many of the resulting predicted proteins coming from different contigs within a species were completely identical along their entire length. Furthermore, we also observed many contigs whose predicted proteins were a subset of the predicted proteins from another contig. For example, contig 1 could have predicted proteins A and B, and contig 2 could have two predicted proteins exactly matching A and B, and a third predicted protein C. Using a custom Perl script, we removed both types of redundancy (exact matches and subsets) from the data set of predicted proteins, and we also removed the contigs from which they were predicted. This process eliminated 60,063 contigs from the decontaminated set, leaving 87,805 contigs remaining, from which 29,482 proteins were predicted.

#### Measurement of expression levels and elimination of noise transcripts

To estimate expression levels, we remapped sequence reads to the decontaminated, non-redundant, Tandem Repeats masked contigs using the Burroughs-Wheeler Aligner, BWA, version 0.7.5a [56]. We ran BWA “mem” with the “-a” option to report all equally best hits. All other BWA parameter values were set to their defaults. We converted BWA output to BAM format using SAMtools version 0.1.18 [57]. We then ran eXpress version 1.4.0 [58] on the resulting BAM files with the option “–rf-stranded” (for strand-specific reads) in order to estimate expression levels, in FPKM, for each contig. All other parameter values were left at their defaults. We examined the distribution of FPKM values across contigs (data not shown) and found the peak of the distribution at values near 1, with steep decreases in the number of contigs at values two orders of magnitude both lower and higher. Therefore, we chose an extremely conservative noise threshold two orders of magnitude below the peak, at FPKM 0.01. We found that 1834 contigs had FPKM values below our noise level of 0.01, and so we eliminated them to produce our final set of 85,971 contigs and 29,154 corresponding predicted proteins.

### Hydroxyurea treatment and RNAseq

Hydroxyurea (HU) treatment decreases the production of deoxyribonulcleotides, leading to cell cycle arrest during S-phase [59]. Control and HU-treated gemmules were grown on 22-mm glass coverslips in six-well culture plate format (10 gemmules per well), with three biological replicates corresponding to Em-CO, Em-BTL, and Em-NR. To ensure HU was applied immediately prior to choanocyte differentiation (i.e., to limit more widespread effects than just choanocyte differentiation) we cultured an “indicator sponge” 1 day prior to starting control and HU-treated sponge cultures. As soon as choanocytes were detected in the indicator sponges (by incubation with India Ink and by microscopic examination), HU (100 µg/mL) was applied to the experimental treatment group, whereas the control groups were untreated. The culture medium (with or without HU) was replaced daily in both the control and HU-treatment groups. After 3 days of HU treatment, choanocytes had not yet developed in any but the control sponges, which were fully differentiated. At this point, phenotypes were documented and tissue was collected for RNA isolation.

RNA was isolated from HU and control sponges using 1 mL Trizol reagent per well, and concentration and quality were analyzed as described above. Total RNA samples were provided to the Genomics and Mircroarray Core Facility (University of Colorado Denver) for Oligo(dT)-selection of mRNA and Illumina library preparation. A total of 6 libraries (three biological replicates in each condition) were multiplexed and sequenced (Hiseq 2000, single-end, 50-bp reads) in a single flow cell lane.

### Identification of sponge homologs of microvillar genes

In general, sponge homologs of vertebrate microvillar genes were identified through best reciprocal blast and validated by domain prediction using SMART [60, 61]. Fine-scale annotation of sponge myosins was performed by adding *Ephydatia* myosin sequences to a published alignment of annotated sequences from *Amphimedon queenslandica* and *Oscarella carmela* ([62]; Additional file 1: Supplemental Figure 1).

### Differential gene expression analysis in *E. muelleri*

Two independent methods were used to examine differential gene expression in control versus HU-treated samples: Corset (v1.03) combined with EdgeR (v3.2; [63]), versus Kallisto [21] combined with Sleuth [22]. Corset is designed to cluster RNA transcripts that presumably derive from a single genomic DNA locus [64]. Corset-mapped reads were analyzed with experimental groups identified (-g) to more efficiently split differentially expressed paralogs. Differential gene expression analysis was conducted on Corset count data using edgeR, a bioconductor package in R [63]. Count data were filtered to remove transcripts with fewer than 1 cpm in at least three samples; this approach is recommended by the EdgeR manual. Data were normalized and GLMTagwise dispersion estimated. GLM testing was conducted using a design matrix to correct for batch effects (~location + treatment), and transcripts were considered to be differentially expressed if they had an FDR corrected (Benjamini–Hochberg) *p* value <0.05 and |log FC| > 1.

Kallisto count data were analyzed by Sleuth using default parameters and results visualized using Shiny v 0.13.0 (http://shiny.rstudio.com). Transcripts were considered to be differentially expressed if they had an FDR corrected (Bonferroni) *p* < 0.05 and |log FC| > 1.

Differentially expressed transcripts were annotated to reflect whether they were less expressed in EdgeR, Sleuth, or both analyses. We ranked transcripts according to three evidence categories: (I) significantly differentially expressed in each of EdgeR and Sleuth analysis of *E. muelleri* (this study), and a published EdgeR analysis of choanocyte expression in *E. fluviatilis* [49]; (II) significantly differentially expressed in either EdgeR or Sleuth, and corroborated by the published *E. fluviatilis* study; (III) evidence for choanocyte expression from *E. fluviatilis*, but no corroborative support for differential expression in choanocytes between *E. muelleri* and *E. fluviatilis.*

## Additional files

10.1186/s13227-016-0050-x Neighbor-joining analysis to cluster Myosins from *Ephydatia* with previously annotated Myosins from *Amphimedon* and *Oscarella* [62].
10.1186/s13227-016-0050-x Expression data for microvillar genes homologs in both *Ephydatia* species. Summary of gene expression results for microvillar genes in *Ephydatia muelleri* and *Ephydatia fluviatilis* [data from 49].
